# Prevalence and Clinical Characteristics of Supernumerary Teeth in a Pediatric Clinical Population: A Retrospective Observational Study

**DOI:** 10.3390/jcm15218488

**Published:** 2026-11-01

**Authors:** Ana Catarina Oliveira, Inês Ferreira, Sofia Sousa-Santos, Beatriz dos Santos, Mariana Azevedo, Primavera Sousa-Santos, Aline Gonçalves, Selma Pascoal, Teresa Vale

**Affiliations:** 1 UNIPRO—Oral Pathology and Rehabilitation Research Unit, CESPU University, 4585-116 Gandra, Portugal; a31643@alunos.cespu.pt (I.F.); sofia.santos@iucs.cespu.pt (S.S.-S.); mariana.azevedo@saude.cespu.pt (M.A.); primavera.santos@iucs.cespu.pt (P.S.-S.); aline.goncalves@iucs.cespu.pt (A.G.); selma.pascoal@iucs.cespu.pt (S.P.); teresa.vale@iucs.cespu.pt (T.V.); 2 Stomatology Department, University Local Health Unit São Joao, 4200-319 Porto, Portugal; beatrizmarianasantos@hotmail.com

**Keywords:** supernumerary teeth, hyperdontia, mesiodens, pediatric dentistry, orthopantomography, prevalence

## Abstract

**Background/Objectives**: Hyperdontia is a dental developmental anomaly frequently associated with clinical complications such as delayed eruption, diastemas, and root resorption. This retrospective observational study aimed to evaluate the prevalence and clinical characteristics of supernumerary teeth in a Portuguese pediatric clinical population. **Methods**: Analyzing orthopantomograms from 1992 patients across two university clinics between 2016 and 2026, demographic and clinical variables were evaluated. The inclusion criteria comprised high-quality, accessible radiographs obtained within the last 10 years from patients aged between 5 and 18 years at the time of the examination. **Results**: The overall prevalence of supernumerary teeth was 1.66%. The mesiodens was the most common type, exclusively maxillary, predominantly conical, and highly impacted (86.7%). A non-significant male predilection was observed. The overall mean age at diagnosis for mesiodens was 9.87 ± 3.98 years. Posterior anomalies presented negligible prevalence (≤0.4%). **Conclusions**: These findings show the mesiodens as the primary supernumerary tooth. The high impaction rate at diagnosis highlights the diagnostic value of panoramic radiography when clinically indicated, supporting timely identification and individualized treatment planning.

## 1. Introduction

Dental development is a complex process that begins during intrauterine life [1]. Any interference in this process can lead to anomalies in number, size, or position, such as supernumerary teeth (hyperdontia), which arise from extra proliferation of the dental lamina or a division of the tooth germ [2].

Supernumerary teeth are defined as teeth that emerge sporadically more than the normal dental formula. They can occur in both the primary dentition, with a prevalence ranging from 0.3% to 0.6% according to the literature [3,4], and the permanent dentition, where the prevalence increases to 0.5–3.8%. The maxilla is the most affected location, particularly the anterior region [4].

The presence of supernumerary teeth can significantly affect the development and eruption of adjacent teeth, resulting in complications such as impaction, dental crowding, root resorption, or the formation of dentigerous cysts. Furthermore, they can impact both dental esthetics and masticatory function, thereby necessitating individualized diagnosis and treatment planning [5,6].

The etiology of hyperdontia is considered multifactorial, involving both genetic and environmental factors [1,7]. Hyperactivity of the dental lamina is the most widely accepted theory, although theories of tooth germ dichotomy and atavism have also been suggested [1,2,6,8,9,10].

Among the various types of supernumerary teeth, the mesiodens stands out as the most common and clinically relevant [1,10]. It can present unilaterally or bilaterally [2,8,9,11,12]. A mesiodens is characteristically a supernumerary tooth located in the midline of the maxilla, typically between the maxillary central incisors [1,12,13].

The literature indicates that the prevalence of mesiodens ranges from 0.15% to 1.9%, with a higher frequency observed in males (a male-to-female ratio of approximately 2:1) [12]. However, genetic and environmental variations can lead to differences in prevalence across distinct populations [11,12,14].

Previous studies conducted in Portuguese clinical populations have included broader age ranges or focused on dental anomalies more generally, limiting the availability of data specifically addressing supernumerary teeth in pediatric populations. Therefore, further research focusing exclusively on pediatric patients is needed to better characterize the prevalence and radiographic characteristics of supernumerary teeth in this age group [15,16].

Although many mesiodens cases remain asymptomatic, their presence can cause significant local complications, including delayed eruption, dental crowding, midline diastemas, malocclusion, root resorption, or cystic changes [6,8,10,12,17,18].

The diagnosis of mesiodens relies heavily on radiographic examination. Cone-beam computed tomography (CBCT) is considered the most accurate method for establishing the three-dimensional location and relationship with adjacent structures, effectively complementing conventional radiographs such as orthopantomograms [6,17,18,19,20,21].

The management of mesiodens must be meticulously personalized, considering the patient’s age, the tooth’s position, and its impact on the adjacent dentition [14]. While surgical extraction is generally recommended to prevent complications like delayed eruption and malocclusion, there is no universal consensus regarding the ideal timing for intervention [12]. Early extraction may facilitate the normal spontaneous eruption of the incisors and reduce surgical complexity, but it carries a risk of damaging developing adjacent structures [6]. Conversely, late removal mitigates these risks but may complicate the surgical procedure and increase the likelihood of established complications. In asymptomatic cases or those with challenging surgical access, clinical and radiographic monitoring can be a viable alternative [6,22].

This retrospective study aimed to determine the prevalence, demographic distribution, and clinical presentation of supernumerary teeth in a Portuguese pediatric clinical population.

## 2. Materials and Methods

### 2.1. Study Design and Ethical Considerations

This was a descriptive and retrospective observational study based on the analysis of pre-existing two-dimensional radiographic records (orthopantomograms) from two university dental clinics: Dr. Filinto Baptista University Clinic (Gandra, Portugal) and Matosinhos University Dental Clinic (Matosinhos, Portugal).

The study was conducted in accordance with the guidelines of the Declaration of Helsinki and was approved by the Ethics Committee of IUCS-CESPU (protocol code: 40/CE-IUCS/2025, approval date: 27 January 2026). Due to the retrospective nature of the study design and the exclusive use of previously anonymized clinical data and radiographic images, the requirement for informed consent was formally waived by the Ethics Committee. Ethical approval was obtained before the initiation of data collection.

### 2.2. Sample Selection and Inclusion Criteria

The source population comprised all pediatric patients treated at the Dr. Filinto Baptista University Clinic (Gandra, Portugal) and the Matosinhos University Dental Clinic (Matosinhos, Portugal) for whom orthopantomographic records were available during the study period. Radiographic records obtained between 2016 and 2026 were all retrospectively reviewed. After application of the predefined eligibility criteria, a final sample of 1992 pediatric patients was included in the study. Of the 1992 included pediatric patients, 1455 were evaluated at the Dr. Filinto Baptista University Clinic (Gandra, Portugal) and 537 at the Matosinhos University Clinic (Matosinhos, Portugal).

The inclusion criteria were: (1) availability of an orthopantomogram of adequate diagnostic quality, free from distortions or artifacts that could compromise visualization of the dental structures; (2) patient age between 5 and 18 years at the time of the radiographic examination; and (3) radiographic examination performed within the predefined 10-year study period (2016–2026).

The exclusion criteria were: (1) repeated radiographic examinations from the same patient; (2) patients with syndromes or systemic conditions known to be associated with hyperdontia; and (3) patients with a previous history of extraction of supernumerary teeth.

The individual patient was considered the unit of analysis, and each patient was included only once. When multiple orthopantomograms were available for the same patient, the first radiograph recorded in the patient’s individual clinical file was selected for analysis. No patient received treatment at both participating centers; therefore, no duplicate records between centers were identified.

### 2.3. Data Collection

Radiographic evaluation and data extraction were performed using EasyDent4 (EWOO Technologies Co., Ltd., Suwon, Republic of Korea). The panoramic radiographs included in the study had been obtained as part of clinical assessment and treatment planning at the two participating university dental clinics, according to the individual diagnostic needs of each patient; no radiographs were acquired specifically for the purposes of this study. Radiographic records obtained between 2016 and 2026 were all retrospectively reviewed. Radiographic quality was considered adequate when the image allowed clear visualization of the maxillary and mandibular dental arches and reliable assessment of dental number, location, morphology, and eruption status, without relevant positioning errors, motion artifacts, image distortion, or other technical limitations that could compromise diagnostic interpretation. Radiographs not fulfilling these criteria were excluded from the analysis.

The two operators (odontopediatric specialists) reviewed and agreed on the diagnostic criteria and classification system before initiating the radiographic assessment. Each orthopantomogram was evaluated independently by both examiners at two separate time points. Any discrepancies were subsequently reviewed jointly and resolved by consensus.

### 2.4. Operational Definitions

The operational definitions used for the radiographic classification of supernumerary teeth are summarized in Table 1.

### 2.5. Statistical Analysis

The extracted data were initially tabulated and organized into a comprehensive database using Microsoft Excel software version 16.114.3 (Microsoft Corporation, Redmond, WA, USA). Subsequent statistical analysis was performed utilizing IBM^®^ SPSS^®^ Statistics software version 32.0 (IBM Corporation, Armonk, NY, USA). Descriptive statistics were primarily employed to characterize the study sample. Categorical variables were described using absolute and relative frequencies (percentages), while continuous variables were expressed as means accompanied by their respective standard deviations (SD). Inferential statistics were applied to assess relationships between variables. For subgroups with very small sample sizes, inferential statistical comparisons were not performed, and the findings were reported descriptively. The Chi-square test or Fisher’s Exact test was used for categorical variables (e.g., gender differences), and the independent samples t-test was utilized to compare continuous variables (e.g., age at diagnosis) between groups. The significance level was set at *p* < 0.05 for all analyses.

## 3. Results

### 3.1. Study Population Demographics

A total of 1992 pediatric patient records were analyzed in this retrospective study, covering a ten-year period (2016–2026). The overall sample presented a mean age of 11.92 ± 4.06 years at the time of the radiographic diagnosis. The age distribution of the sample approximated a normal distribution (Figure 1).

Regarding gender distribution, a slight male predominance was observed, comprising 54.4% of the sample (*n* = 1084), while females represented 45.6% (*n* = 908). Concerning the stage of dental development, most of the patients were in the permanent dentition phase (56.9%), followed by the mixed dentition phase (41.7%), with the primary dentition representing a minority (1.4%).

### 3.2. Overall Prevalence of Supernumerary Teeth

A total of 33 of the 1992 pediatric patients examined presented at least one supernumerary tooth (Table 2). Therefore, the patient-level prevalence of hyperdontia was 1.66% (33/1992; 95% CI: 1.18–2.32%). Regarding the dentition stage among the affected individuals, 21 patients were in the permanent dentition phase and 12 were in the mixed dentition phase. Furthermore, 6 of the 33 affected patients presented with more than one supernumerary tooth. The denominator used for this prevalence estimate was the total number of patients included in the study, with each patient counted once regardless of the number of supernumerary teeth identified.

Among the 33 affected patients, 23 were male and 10 were female. Hyperdontia was identified in 2.1% of male patients (23/1084; 95% CI: 1.42–3.16%) and 1.1% of female patients (10/908; 95% CI: 0.60–2.02%). Although the proportion was numerically higher among males, the association between sex and hyperdontia did not reach statistical significance (*p* = 0.080) according to Fisher’s test.

### 3.3. Clinical and Radiographic Characterization

Although prevalence was calculated at the patient level, detailed analysis revealed that a subset of patients presented with multiple supernumerary teeth, as summarized in Table 3.

#### 3.3.1. Mesiodens

The mesiodens was the most prevalent anatomical typology, representing 45.5% (*n* = 15) of all hyperdontia cases. Clinically, 100% of the identified mesiodens were in the maxilla, strictly adhering to their anatomical definition. Morphological evaluation demonstrated a predominantly conical shape, present in 80.0% of the mesiodens (*n* = 12), with the remaining 20.0% (*n* = 3) presenting a tuberculate morphology.

A critical finding was the high rate of radiographic impaction: 86.7% of the mesiodens (*n* = 13) were impacted at the time of diagnosis. Regarding sex distribution, 12 of the 15 patients with mesiodens were male and 3 were female, corresponding to a male-to-female ratio of 4:1, as shown in Table 4. Given the small number of cases, this finding should be interpreted descriptively and does not establish a statistically demonstrated sex difference (*p* = 0.066) according to Fisher’s test.

#### 3.3.2. Posterior and Other Supernumerary Anomalies

Anomalies located outside the anterior maxilla presented drastically lower prevalences. Distomolar teeth (distodens) were the second most common anomaly, representing 24.2% of the affected cases (*n* = 8), with an almost exclusive location in the maxilla. Supplemental premolars accounted for 21.2% (*n* = 7) of the cases and presented a more heterogeneous anatomical distribution, frequently involving multiple quadrants and showing a slight predilection for the mandible. Paramolars were exceptionally rare, with only a single case (3.0%) identified. Finally, two atypical cases (6.1%) of supplemental teeth located in the anterior region of the mandible were also recorded.

## 4. Discussion

The primary objective of the present study was to determine the prevalence and characterize the clinical presentation of supernumerary teeth in a pediatric clinical population, utilizing a sample of 1992 patients.

These findings agree with current scientific evidence. In a study published by *Chen* et al. (2019) [5], the prevalence of supernumerary teeth was estimated to range from 0.5% to 3.8% in the permanent dentition, in contrast to the lower rates of 0.3% to 0.6% recorded in the primary dentition. Since the population of the present study falls within the 5-to-18 age range, encompassing the primary, mixed, and permanent dentition phases, the rate of 1.66% verified at the clinics falls within these reference values. This fact confirms the rarity of these numerical anomalies.

Regarding typology, the mesiodens stood out as the most prevalent supernumerary tooth in the present sample, being widely in agreement with the scientific literature that positions it as the most frequent numerical anomaly [13]. The totality of the diagnosed cases (100%) was in the maxilla, especially in the midline region. This anatomical preference for the premaxilla is a classic finding, frequently justified in the literature by the hyperactivity of the dental lamina in this specific region during embryogenesis [6,12].

From a morphological standpoint, a clear predominance of the conical shape (80.0%) was observed, followed by the tuberculate (20.0%) form. Simultaneously, a high retention rate was verified, with 86.7% of these supernumerary teeth presenting in an impacted state at the time of radiographic diagnosis. These findings are consistent with the existing literature, which reports a predominance of conical morphology and a high frequency of impaction among mesiodens [10]. According to scientific evidence, the conical morphology, together with an inverted or transverse axis of development, constitutes a mechanical obstacle that impedes the normal physiological eruptive pathway [23]. In this way, the results of our sample mirror the behavior pattern for the mesiodens, justifying the high percentage of clinical retention observed.

Regarding sexual dimorphism, the present study evidenced a susceptibility in the male gender, establishing a 4:1 ratio compared to the female gender. This disparity toward the male gender appears higher than some proportions commonly mentioned in the literature, although it aligns with the global trend of a higher prevalence of mesiodens in male individuals [2,10,11,24,25]. However, it is fundamental to interpret this statistical finding with caution. Despite the obvious clinical discrepancy observed in the sample, the analysis did not reach statistical significance (*p* = 0.066). This absence of statistical association is justified by the reduced number of affected patients, which limits the power of the test to detect significant differences between genders. Consequently, future studies with larger sample sizes of affected patients are highly recommended to further consolidate the statistical significance of these observed demographic and clinical trends. Multi-center longitudinal studies would be particularly valuable to overcome local sample biases, providing a robust statistical framework capable of validating whether this 4:1 ratio represents a true epidemiological shift or a regional sample fluctuation.

When comparing our findings with the broader international literature [10,11,12], the overall prevalence falls within accepted ranges, but specific clinical presentations, such as our 4:1 male-to-female ratio and the 86.7% impaction rate, exhibit some variance. These discrepancies between studies can be attributed to several fundamental factors. Firstly, population-specific genetic and ethnic characteristics strongly dictate the morphological expression and distribution of supernumerary teeth. Secondly, methodological disparities, such as the age ranges of the studied cohorts, the strictness of inclusion criteria, and the imaging modalities used (e.g., two-dimensional orthopantomograms versus 3D CBCT), substantially impact the reported data. Finally, the sampling setting plays a crucial role. Because our data originates from university dental clinics, it is subject to a natural referral bias. Such settings typically concentrate more complex clinical scenarios, including cases with delayed eruption or severe impaction, which directly explains the exceptionally high rate of impacted mesiodens observed in our specific pediatric sample.

In our sample, mesiodens were radiographically detected at a mean patient age of 9.87 years. The timing of this detection exhibited individual variability. Earlier identifications are in conformity with the literature, which advocates for early screening during the transition to the mixed dentition, frequently motivated by aesthetic complaints or delays in the eruption of the central incisors. Conversely, later detections reflect a delayed initial dental consultation by these patients, combined with the characteristic asymptomatic nature of this type of supernumerary tooth, which often masks its presence until a panoramic radiograph is performed upon medical recommendation [11].

In contrast, the remaining typologies of supernumerary teeth identified in the present study, namely supplemental premolars, distodens, and paramolars, were radiographically detected at older ages. This trend of a later diagnosis is fully justified by the tooth eruption chronology [18,24,26,27,28]. Unlike the mesiodens, which frequently causes early esthetic or occlusal disturbances in the anterior region, these posterior supernumeraries tend to have an asymptomatic development and do not interfere as obviously with the eruption of adjacent teeth. Consequently, their clinical identification frequently occurs accidentally during orthopantomographies [7].

The high impaction rate observed in the present sample (86.7%) strongly reinforces the importance of orthopantomography as the primary diagnostic tool [6,10,11,12]. Because these anomalies are mostly asymptomatic during early development, radiographic screening during the transition to the mixed dentition is an indispensable preventive baseline protocol [12]. Without such proactive screening, these anomalies remain silent dynamic threats, quietly inducing root resorption of adjacent teeth, dentigerous cyst formation, or severe midline deviations before any clinical symptom becomes visible. Should surgical intervention be indicated, this initial two-dimensional (2D) screening effectively paves the way for targeted CBCT to guide precise surgical planning [6,17,18,19,20,21]. Ultimately, the failure to obtain these timely radiographic diagnoses can lead to clinical problems and compromise the normal development of the pediatric patient. The functional and esthetic impact of this anomaly can be observed in a clinical case report published in the literature, which describes a clinical situation of prolonged retention and a late diagnosis of a mesiodens resulting in common sequelae such as ectopic eruption of the central incisor and midline diastema [29].

Consequently, avoiding late diagnosis is crucial to prevent prolonged retention and complex sequelae, such as ectopic eruption and midline diastemas [29]. However, the early detection advocated by our findings does not dictate immediate surgical removal. The management of mesiodens remains highly individualized [6,14], requiring a careful balance between the risks of early extraction (potential damage to developing structures) [6] and the complications of delayed removal (increased surgical difficulty) [12]. Therefore, early radiographic screening provides the clinician with the crucial timeframe needed for meticulous monitoring, allowing for the selection of the ideal interventional window [6,22].

Several limitations of the present study should be acknowledged. First, its retrospective design and clinic-based sampling may introduce selection and referral bias, as only patients who underwent panoramic radiography for clinical reasons were included. Second, the use of two-dimensional panoramic imaging may limit the precise assessment of the position and morphology of some supernumerary teeth. In addition, although the overall sample was large, the number of patients with hyperdontia was limited, and some individual subgroups contained very few cases, reducing the precision of subgroup estimates and limiting inferential analyses. Family history information was not consistently recorded in the retrospective clinical records. The wide age range and heterogeneous dentition stages may also have influenced the timing and detection of the anomalies. Taken together, these factors should be considered when interpreting the findings and limit their generalizability to the broader Portuguese pediatric population. The scarcity of national literature in the field of pediatric dentistry highlights the gap that this work aims to fill, underscoring the novelty and epidemiological value of our study.

## 5. Conclusions

The prevalence of supernumerary teeth in the evaluated clinic-based pediatric sample was 1.66%, in line with previous reports. Mesiodens was the most frequent anatomical type and was predominantly located in the maxilla, with a conical morphology and a high rate of radiographic impaction. A higher proportion of cases was observed among males; however, no statistically significant association with sex was demonstrated. Mesiodens tended to be identified at a younger age than posterior supernumerary anomalies.

## Figures and Tables

**Figure 1:**
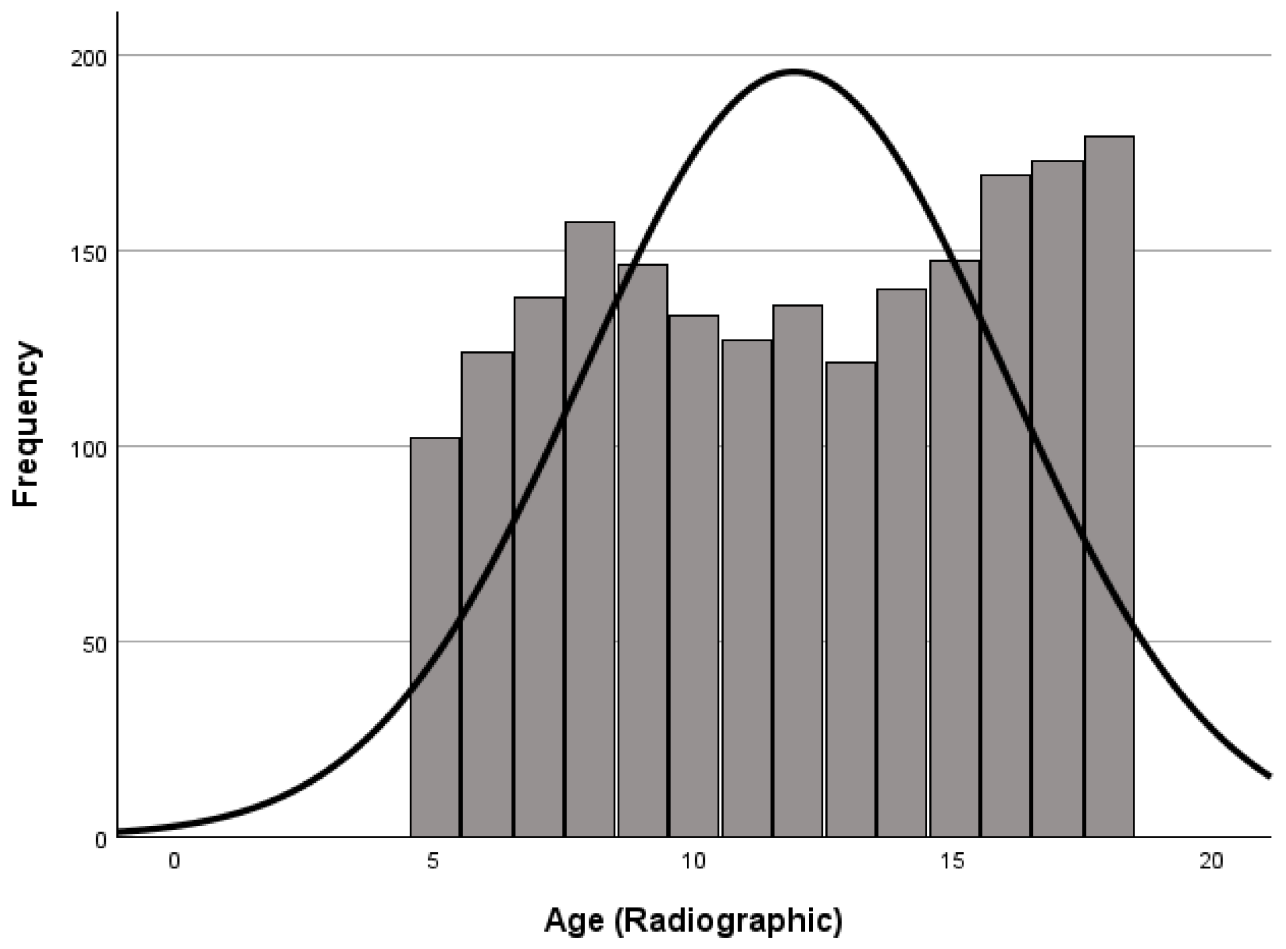
Distribution of the total sample by age (*n* = 1992).

**Table 1:** Operational definitions used for the radiographic classification of supernumerary teeth.

	Classification	Operational Definition
Supranumerary types	Mesiodens	Supernumerary tooth located in the anterior maxillary midline region, typically between the maxillary central incisors.
	Paramolar	Supernumerary tooth located adjacent to the buccal or palatal aspect of a molar.
	Distomolar	Supernumerary tooth located distal to the most posterior molar.
	Supplemental tooth	Supernumerary tooth morphologically resembling a normal tooth in the corresponding region of the dental arch.
Morphology	Conical	Supernumerary tooth presenting a small, peg-shaped or cone-shaped radiographic appearance.
	Tuberculate	Supernumerary tooth presenting a broader, barrel-shaped appearance, frequently with more than one cusp or tubercle.
Location	Dental arch	Classified as maxillary or mandibular according to the anatomical arch in which the supernumerary tooth was identified.
	Region	Classified as anterior or posterior according to its anatomical position within the corresponding dental arch.
Impaction	Impacted	Supernumerary tooth remaining intraosseous, with no radiographic evidence of eruption into the dental arch at the time of examination.
	Non-impacted	Supernumerary tooth showing radiographic evidence of eruption into, or emergence within, the dental arch.

**Table 2:** Overall prevalence of supernumerary teeth.

Presence of Supernumerary Teeth	Frequency		Percentage
Absent	1959		98.34
Present	33		1.66
**Typology**	**n (%)**	**Location**	**Age at Diagnosis (Mean/Range)**
Mesiodens	15 (45.5%)	15 Maxilla	9.87 ± 3.98
Distodens	8 (24.2%)	7 Maxilla, 1 Mandible	15.25 ± 2.49
Supplementary Premolars	7 (21.2%)	3 Maxilla, 4 Mandible	12.00 ± 3.56
Paramolars	1 (3.0%)	1 Maxilla	14.00
Other Atypical	2 (6.1%)	2 Mandible	10.00
Total	1992		100.00

**Table 3:** Breakdown of patients presenting with multiple supernumerary teeth.

Type of Supernumerary Tooth	Number of Affected Patients	Anatomical Distribution
Mesiodens	1	2 Mesiodens
Distodens	3	2 Distodens per patient (located in the first and second quadrants)
Supplementary Premolars	2	3 Supplemental Premolars (located in the first, second and third quadrants) and 2 Supplemental Premolars (located in the third and fourth quadrants)
Total	6	-

**Table 4:** Clinical and radiographic characteristics of mesiodens cases (*n* = 15).

Presence of Mesiodens Teeth		Frequency	Percentage
Location	Maxilla	15	100
	Mandibula	0	0
Morphology	Conical	12	80
	Tuberculate	3	20
Eruption Status	Impacted	13	86.7
	Non-Impacted	2	13.3
Gender	Male (1.084)	12	1.11
	Female (908)	3	0.33

## Data Availability

The data presented in this study are available on request from the corresponding author. The data are not publicly available due to privacy and ethical restrictions related to pediatric patient clinical records.

## Abbreviations

The following abbreviations are used in this manuscript:

2D	Two-dimensional
CBCT	Cone-beam computed tomography
IUCS-CESPU	Instituto Universitário de Ciências da Saúde-CESPU
